# Prepuberal Stimulation of 5-HT7-R by LP-211 in a Rat Model of Hyper-Activity and Attention-Deficit: Permanent Effects on Attention, Brain Amino Acids and Synaptic Markers in the Fronto-Striatal Interface

**DOI:** 10.1371/journal.pone.0083003

**Published:** 2014-04-07

**Authors:** Lucia A. Ruocco, Concetta Treno, Ugo A. Gironi Carnevale, Claudio Arra, Gianpiero Boatto, Maria Nieddu, Cristina Pagano, Placido Illiano, Fabiana Barbato, Angela Tino, Ezio Carboni, Giovanni Laviola, Enza Lacivita, Marcello Leopoldo, Walter Adriani, Adolfo G. Sadile

**Affiliations:** 1 Department of Experimental Medicine, School of Medicine, II University of Naples, Napoli, Italy; 2 Animal facility, National Cancer Institute, Napoli, Italy; 3 Department of Chemistry and Pharmacy, University of Sassari, Sassari, Italy; 4 Istituto di Cibernetica “Eduardo Caianiello” - CNR, Pozzuoli, Napoli, Italy; 5 Department of Biomedical Sciences, University of Cagliari, Cagliari, Italy; 6 Section of Behavioural Neuroscience, Department of Cell Biology and Neurosciences, Istituto Superiore di Sanità, Roma, Italy; 7 Department of Medicinal Chemistry, University of Bari, Bari, Italy; Radboud University, Netherlands

## Abstract

The cross-talk at the prefronto-striatal interface involves excitatory amino acids, different receptors, transducers and modulators. We investigated long-term effects of a prepuberal, subchronic 5-HT7-R agonist (LP-211) on adult behaviour, amino acids and synaptic markers in a model for Attention-Deficit/Hyperactivity Disorder (ADHD). Naples High Excitability rats (NHE) and their Random Bred controls (NRB) were daily treated with LP-211 in the 5th and 6th postnatal week. One month after treatment, these rats were tested for indices of activity, non selective (NSA), selective spatial attention (SSA) and emotionality. The quantity of L-Glutamate (L-Glu), L-Aspartate (L-Asp) and L-Leucine (L-Leu), dopamine transporter (DAT), NMDAR1 subunit and CAMKIIα, were assessed in prefrontal cortex (PFC), dorsal (DS) and ventral striatum (VS), for their role in synaptic transmission, neural plasticity and information processing. Prepuberal LP-211 (at lower dose) reduced horizontal activity and (at higher dose) increased SSA, only for NHE but not in NRB rats. Prepuberal LP-211 increased, in NHE rats, L-Glu in the PFC and L-Asp in the VS (at 0.250 mg/kg dose), whereas (at 0.125 mg/kg dose) it decreased L-Glu and L-Asp in the DS. The L-Glu was decreased, at 0.125 mg/kg, only in the VS of NRB rats. The DAT levels were decreased with the 0.125 mg/kg dose (in the PFC), and increased with the 0.250 mg/kg dose (in the VS), significantly for NHE rats. The basal NMDAR1 level was higher in the PFC of NHE than NRB rats; LP-211 treatment (at 0.125 mg/kg dose) decreased NMDAR1 in the VS of NRB rats. This study represents a starting point about the impact of developmental 5-HT7-R activation on neuro-physiology of attentive processes, executive functions and their neural substrates.

## Introduction

The interface between the prefrontal cortex (PFC) and the striata represents the neural substrate for the parallel processing of cognitive and non-cognitive information [1], [2]. Therefore, this neural site has been the target of neurophysiological and imaging studies in relation to neuropsychiatric problems [3], [4]. The neurogenetic approach in model systems has been used so far to study complex behaviour [5], [6] and its neural substrates. This approach will likely lead to a better understanding of neuropsychiatric problems such as Attention-Deficit Hyperactivity Disorder (ADHD), Autism, Schizophrenia and Depression.

In the mammalian brain, the communication between the PFC and the dorsal/ventral striatum involves the amino acid L-Glutamate (L-Glu) acting through different ionotropic [7] and metabotropic receptors, transduction mechanisms and various modulators [8], [9]. The latter include dopamine (DA), norepinephrine (NE), serotonin (5-HT), and histamine. A series of clinical, pharmacological, biochemical and molecular biology studies have supported the “dopamine hypothesis” in the last fifty years [10], [11], which has yielded a wealth of information giving rise to a major knowledge in the field of neurosciences. For instance, in the case of ADHD, DA-ergic psychostimulant drugs like methylphenidate (MPH) and the amphetamines have been largely used. Notwithstanding, the amino acid transmission between the PFC and the striata is modulated by 5-HT that is released by axon terminals of raphe nuclei and may operate through seven receptor families. Among these, the 5-HT7 receptor subtype is the target of LP-211, a newly synthesised selective agonist [12], [13]. It is likely to hypothesise that this receptor could serve a new therapeutic target for ADHD and other neuropsychiatric problems sharing attention deficit.

Therefore, in this manuscript we attempt to investigate long-term effects of a prepuberal, subchronic treatment, by LP-211, on adult behaviour, amino acid transmitters and synaptic markers, using a well documented rat model for ADHD [14]. Animal models for studying ADHD can be of genetic and non-genetic type [15]. The Naples High Excitability (NHE) rat is a classically validated genetic model, which reproduces the mesocortical variant of ADHD [6], [14], [16], [17]. In particular, their profile is characterised by a dysfunctioning mesocortical DA branch with an attention deficit, hyperactivity and altered executive functions. Therefore, adolescent male rats of the Sprague-Dawley-derived NHE line, and their Naples Random-Bred (NRB) [6], [16] controls, received daily exposure to LP-211 (0.0, 0.125, 0.250 or 0.500 mg/kg), from post-natal day 30–31 to 43–44.

By using a multidisciplinary approach, here we show that prepuberal LP-211 yields long lasting changes on adult behaviour, improving spatial attention in a dose dependent manner, associated with modified expression of pre- and post-synaptic markers.

The results of the present experiments may represent an important starting point to explore how selective 5-HT7 receptor stimulation can impact upon the neurophysiology of attentive processes, executive functions and their neural substrates. This may, in turn, lead to a better understanding of developmental neuropsychiatric disorders that share activity, attention and executive function problems.

## Results

### Body weight

To evaluate possible LP-211 developmental-treatment influences on body growth, the body weight was measured in all groups analyzed. There were no differences across groups in NHE and NRB rats. In fact, separate two-way factorial ANOVAs on NHE and NRB rats, with treatment x time (as covariate), showed no main effect for treatment but only for age (time, F = 4111.3 and 832.94; df = 1/26 and 2/45; p<0.0001 for both) with no interaction.

### Long-term behavioural effects of adolescent LP-211 in rats

**Figure 1:**
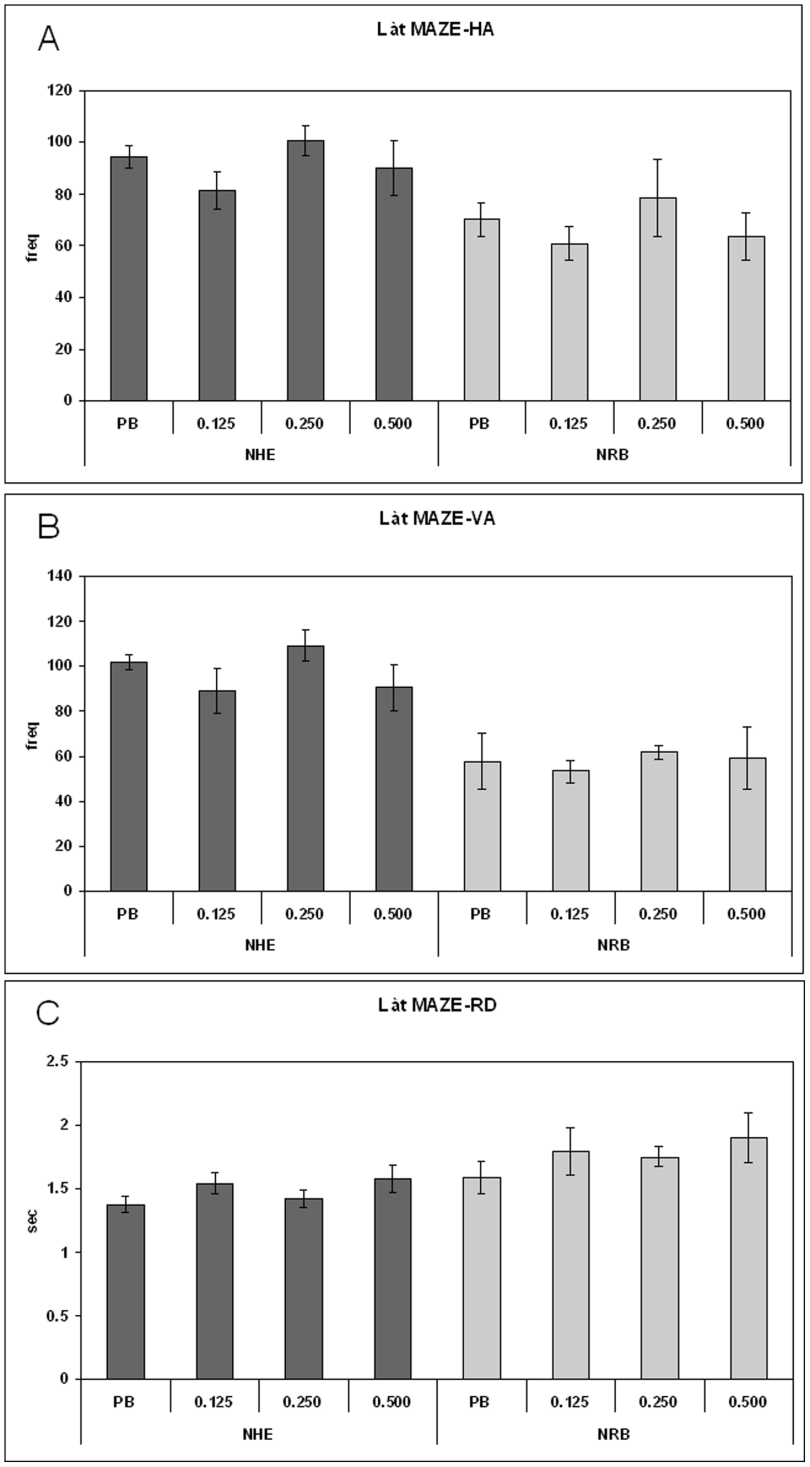
Long term behavioural effects of prepuberal subchronic LP-211 treatment on activity and non selective attention in the Làt maze for adult NHE and NRB rats. The frequency of corner crossings (A), the frequency of rearings (B), and their duration (C) after vehicle or treatment with 0.125, 0.250 or 0.500 mg/kg LP-211 are reported as mean ± SEM, (N = 6/group). For statistical analysis see the Results section.

**Figure 2:**
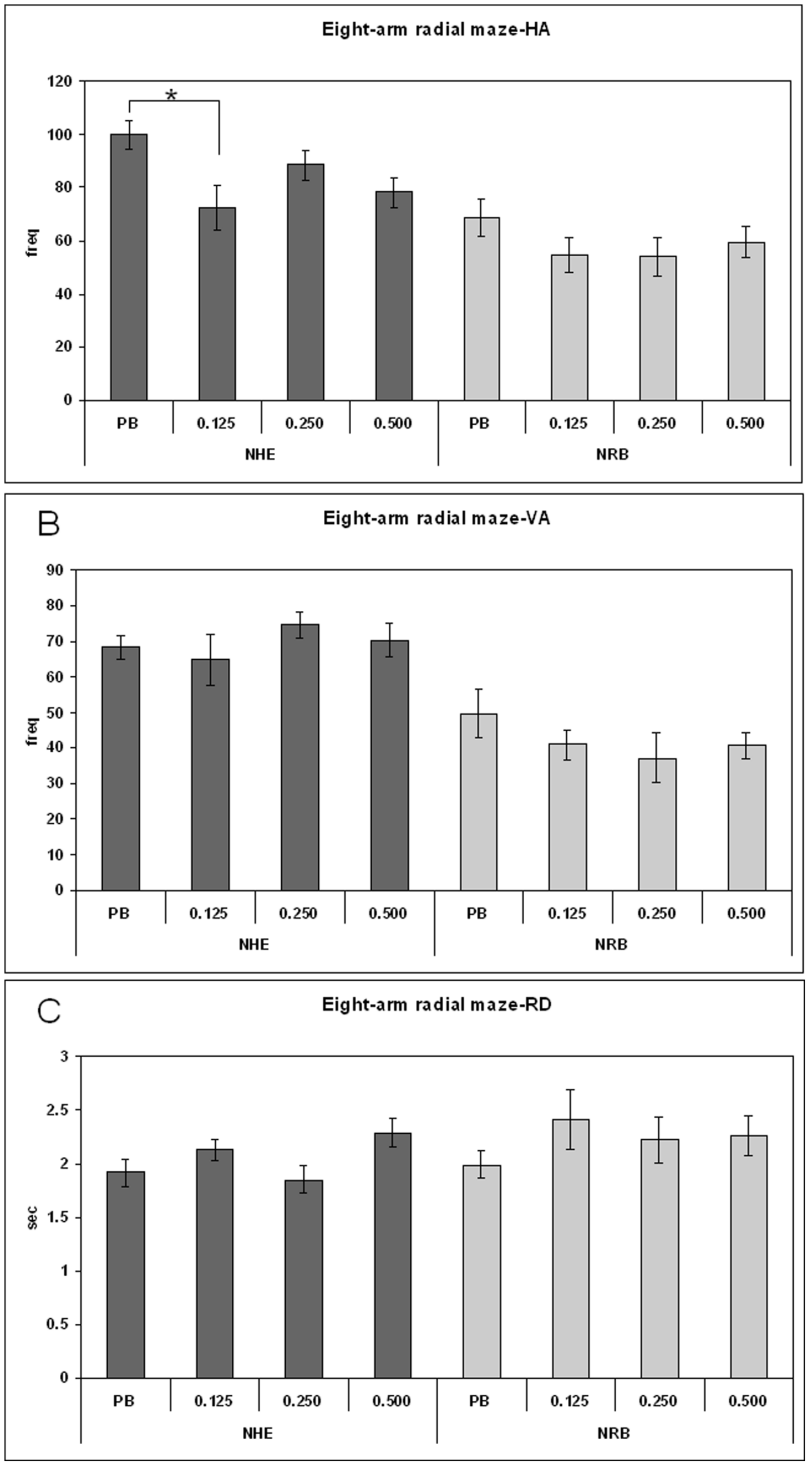
Long term behavioural effects of prepuberal subchronic LP-211 treatment on activity and non selective attention in the Olton maze for adult NHE and NRB rats. The horizontal activity (A), the frequency of rearings (B) and their duration (C) after vehicle or treatment with 0.125, 0.250 or 0.500 mg/kg LP-211 are reported as mean ± SEM, (N = 6/group). For statistical analysis see the Results section (* p<0.01).

Behavioural tests were carried out on young adult rats (pnd 70–75 days) so that long-term effects of prepuberal subchronic LP-211 treatment could be determined. The behavioural tests consisted of short exposure of the rats to spatial novelties paradigms, i.e. Làt and Olton mazes. The main variables were total activity, non selective and selective spatial attention (NSA, SSA), and emotionality index in Broadhurst's terms [18]. Exposure to spatial novelties within a Làt maze (Figure 1A–C) yielded no significant treatment effect for NHE and NRB rats, in separate univariate ANOVAs for indices of activity, i.e. both horizontal activity (HA, panel A) and vertical activity (VA, panel B) as well as rearing duration (RD, panel C). Exposure to spatial novelties within a Olton's eight-arm radial maze shows that LP-211 treatment reduced exploratory HA activity only in the NHE rats (Figure 2A) (one-way ANOVA: effect for treatment (F = 3.56; df = 3/20; *p*<0.033) and for time (F = 4.886; df = 5/110; *p*<0.000) with no treatment per time interaction). The post-hoc LSD test confirmed that the reduction of HA produced by LP-211 was due to the 0.125 mg/kg dose (*p*<0.0064). On the other hand, the vertical activity in both NHE and NRB rats, VA and RD, were not influenced by prepuberal LP-211 treatment (one-way ANOVAs: no significant treatment effect on VA and RD) as was observed in the Làt maze (Figure 2B–C).

**Figure 3:**
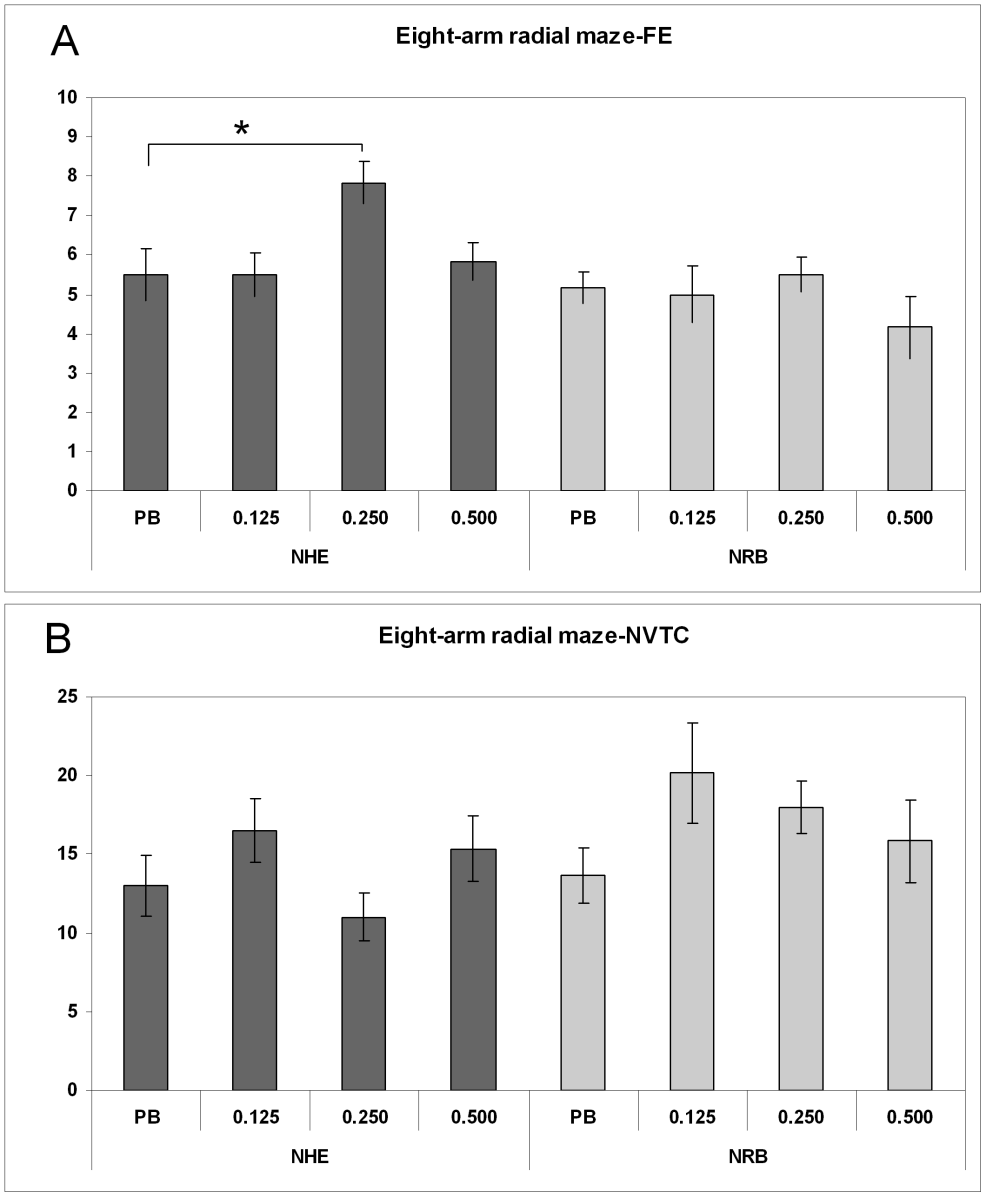
Long term behavioural effects of prepuberal subchronic LP-211 treatment in the Olton maze. Spatial selective attention: the position of first repetitive arm visit (FE; panel A) and number of arm visits before all arms are visited (NVTC; panel B) are reported for the NRB and NHE rats. An efficient rat will explore the eight arms only one time, thus the best score should be 9 for first error, and 8 for number of arm visits to criterion. Data after vehicle or treatment with 0.125, 0.250 or 0.500 mg/kg LP-211 are presented as mean ± SEM, (N = 6/group). For statistical analysis see the Results section (* p<0.01).

For the selective spatial attention (SSA), the two markers of non-reinforced SSA are depicted in Figure 3 panels A and B, respectively. The position of the First repetitive arm Entry (FE) significantly improved only in NHE rats, as demonstrated by one-way ANOVA (F = 3.908; df = 3/20; *p*<0.024). Post-hoc LSD test showed that the higher score was due to the 0.250 mg/kg dose of LP-211 (*p*<0.0087). In contrast, the other parameter, i.e. the total Number of alley Visits needed To Criterion (NVTC), was not different among groups, as demonstrated by one-way ANOVA. The defecation score, used as an emotionality index in Broadhurst terms, was not different across treatment groups and behavioural tasks (Làt and Olton mazes), as demonstrated by separate Kruskal–Wallis tests within the NHE and NRB rats.

### Long-term effects of LP-211 on biochemical parameters in adult rats

It is very well known the role of excitatory amino acids in the synaptic dialogue between the pre-frontal cortex (PFC) and the dorsal/ventral striatum (DS/VS) [19], [20]. L-Glu and L-Asp can play a role in synaptic transmission, neural plasticity and information processing [21]. After the completion of behavioural tests, the various groups of rats were analyzed for the *ex-vivo* levels of the amino acids L-Glu, L-Asp and L-Leucine in the PFC, DS and VS by LC/MS/MS. The choice of L-Asp was based on its hypothesised role in neurotransmission as suggested by D'Aniello et al. [22] and for its proposed involvement in the formation of N-Methyl D-aspartate (NMDA). In fact, L-Asp can be converted into the stereoisomer D-aspartate by the racemase enzyme, then be inserted as such into NMDA by the N-Methyl transferase and act as an agonist at NMDA receptors [22], [23].

**Figure 4:**
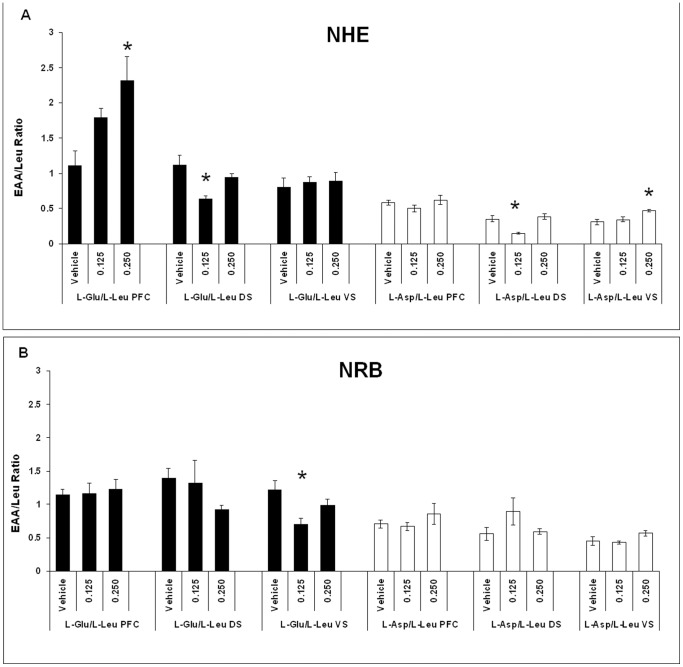
Level of Glu/Leu (black) and Asp/Leu (white) expressed as ratio in PFC, DS and VS of NHE (panel A) and NRB (panel B) rats. Data after vehicle or treatment with 0.125 or 0.250/kg LP-211 are presented as mean ± SEM, (N = 6/group). For statistical analysis see the Results section (* p<0.02).

Excitatory amino acids L-Glu and L-Asp were expressed as a ratio over L-Leu that does not participate into neurotransmission. Moreover, they were measured in the soluble fraction that corresponds to the one sampled by the microdyalisis method [24]. Data pertaining to the forebrain sites are shown (Figure 4A: NHE and Figure 4B: NRB).

#### Prefrontal Cortex

Separate one-way ANOVAs on NHE and NRB rats showed an increased ratio of L-Glu/L-Leu (F = 6.14; df = 2/15, p<0.011) for LP-211 at 0.250 mg/kg dose in the NHE line (Figure 4A). Conversely, no significant changes were observed for any of the doses in NRB rats (Figure 4B). Furthermore, the level of L-Asp/L-Leu did not vary in the PFC of NHE or NRB rats for any of the doses (Figure 4A–B).

#### Dorsal Striatum

In the DS, separate one-way ANOVAs on NHE and NRB rats revealed a decreased L-Glu/L-Leu ratio only for the 0.125 mg/kg dose (F = 7.23; df = 2/15, p<0006) in the NHE line. In contrast, no significant difference was observed in the levels of L-Glu/L-Leu in the NRB line. Furthermore, the L-Asp/L-Leu ratio was also reduced at the 0.125 mg/kg dose (F = 11.50; df = 2/15, p<0.001) in the NHE rats. No significant changes were observed across amino acids and LP-211 doses in the NRB rats (Figure 4B).

#### Ventral Striatum (or nucleus accumbens)

Finally, the levels of L-Glu/L-Leu in the VS of NHE rats showed no significant differences. Separate one-way ANOVAs on NHE and NRB rats demonstrated a reduced L-Glu/L-Leu ratio in the VS for the 0.125 mg/kg dose (F = 6.43; df = 2/15; p<0.010), only in NRB rats (Figure 4B). Moreover, there was an increased L-Asp/L-Leu ratio (F = 7.887, df = 2/14, p<0.005) for the 0.250 mg/kg dose in the NHE line (Figure 4A). The increased L-Glu in the PFC and L-Asp in the VS, produced by the 0.250 mg/kg dose within the NHE line, were the most relevant findings from the point of view of fronto-striatal cross talk.

### Long-term effects of LP-211 on neuronal substrate in adult rats

**Figure 5:**
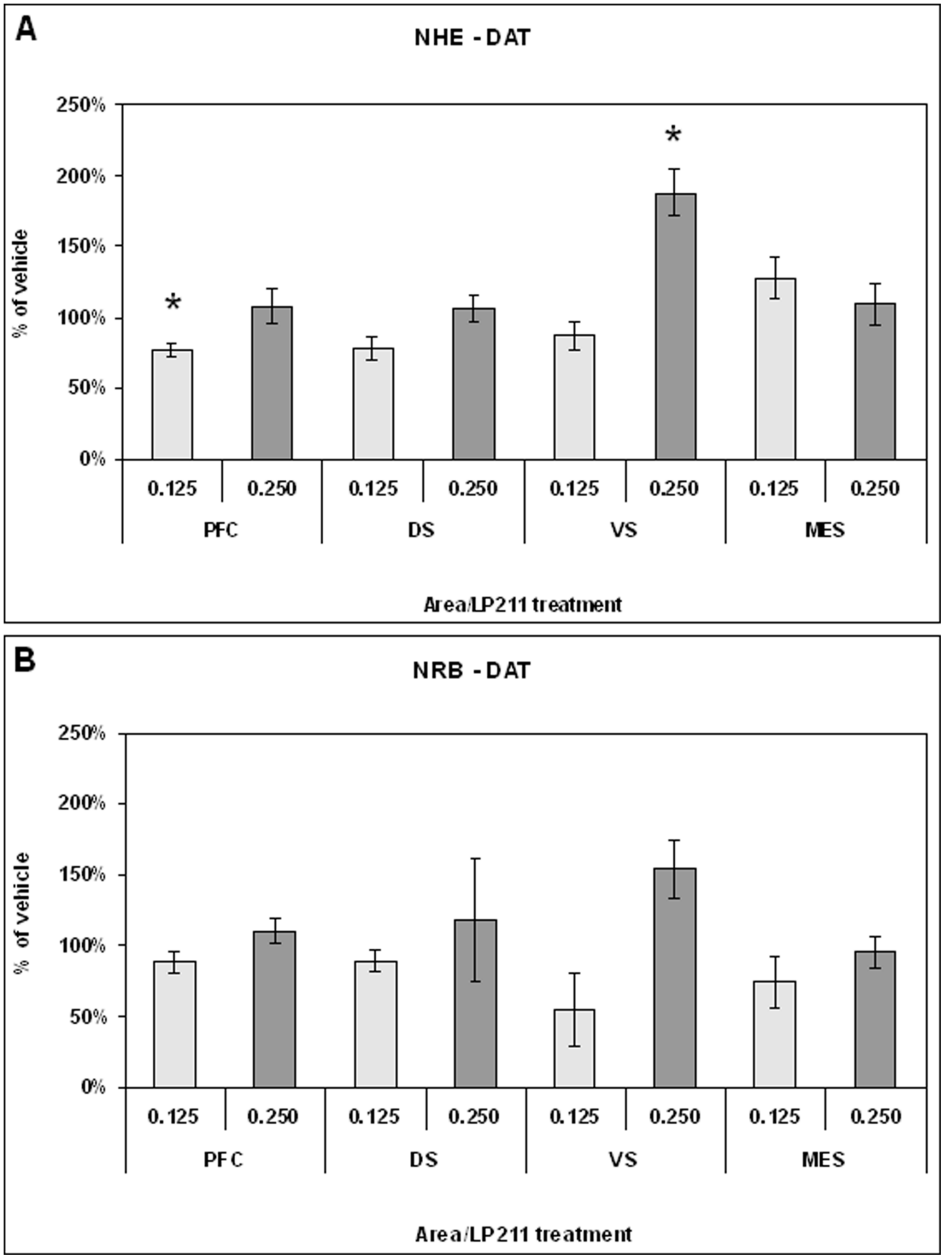
Level of DAT protein measured by western blot in the PFC, DS, VS and ME of NHE (panel A) and NRB (panel B) rats after adolescent treatment with 0.125 or 0.250 mg/kg LP-211. Data are given as % of vehicle response ± standard error. For statistical analysis see the Results section (* p<0.05).

**Figure 6:**
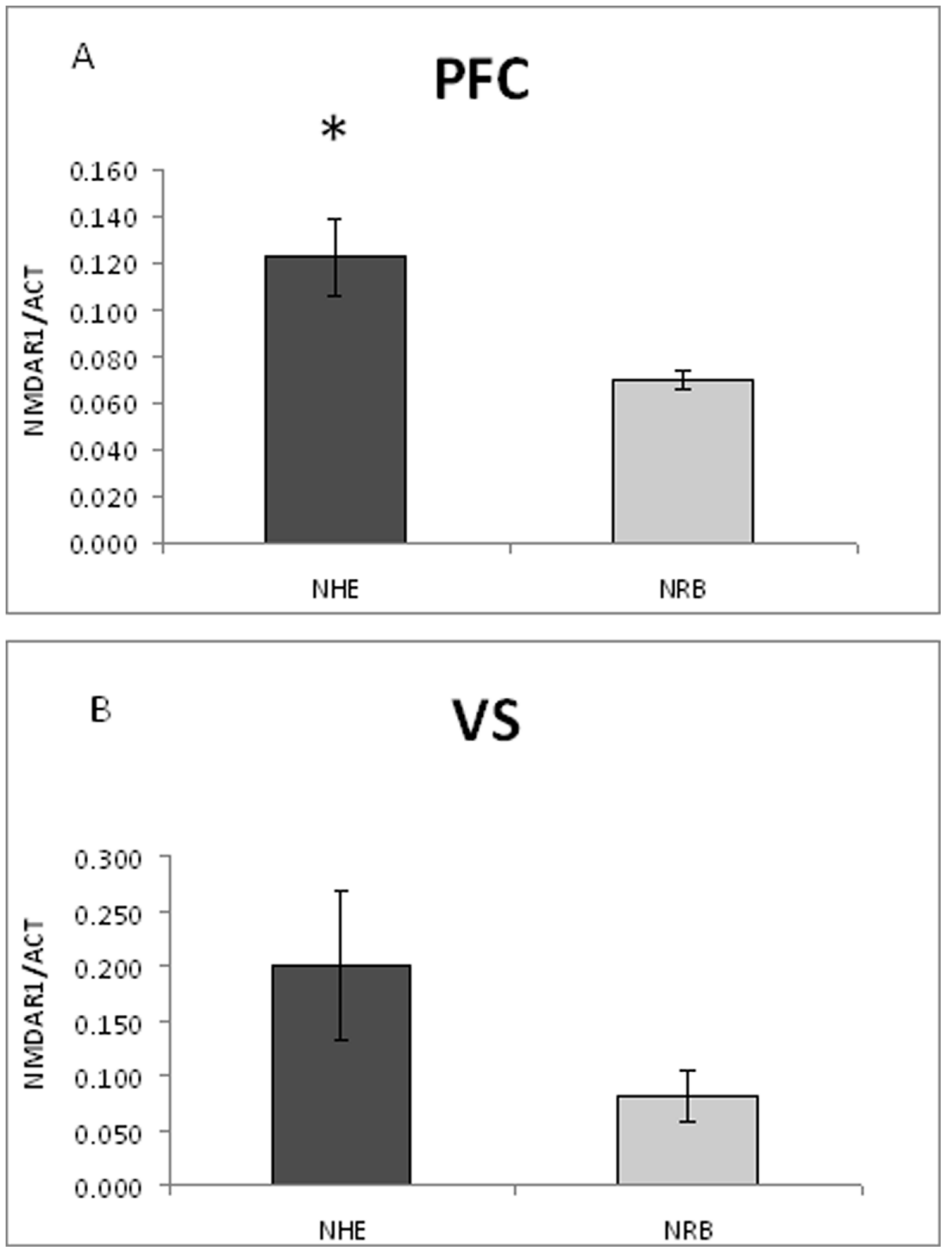
Level of NMDAR1 protein measured in the PFC (panel A) and VS (panel B) of NHE and NRB rats under basal conditions. Data are presented as mean ± standard error. For statistical analysis see the Results section (* p<0.05).

**Figure 7:**
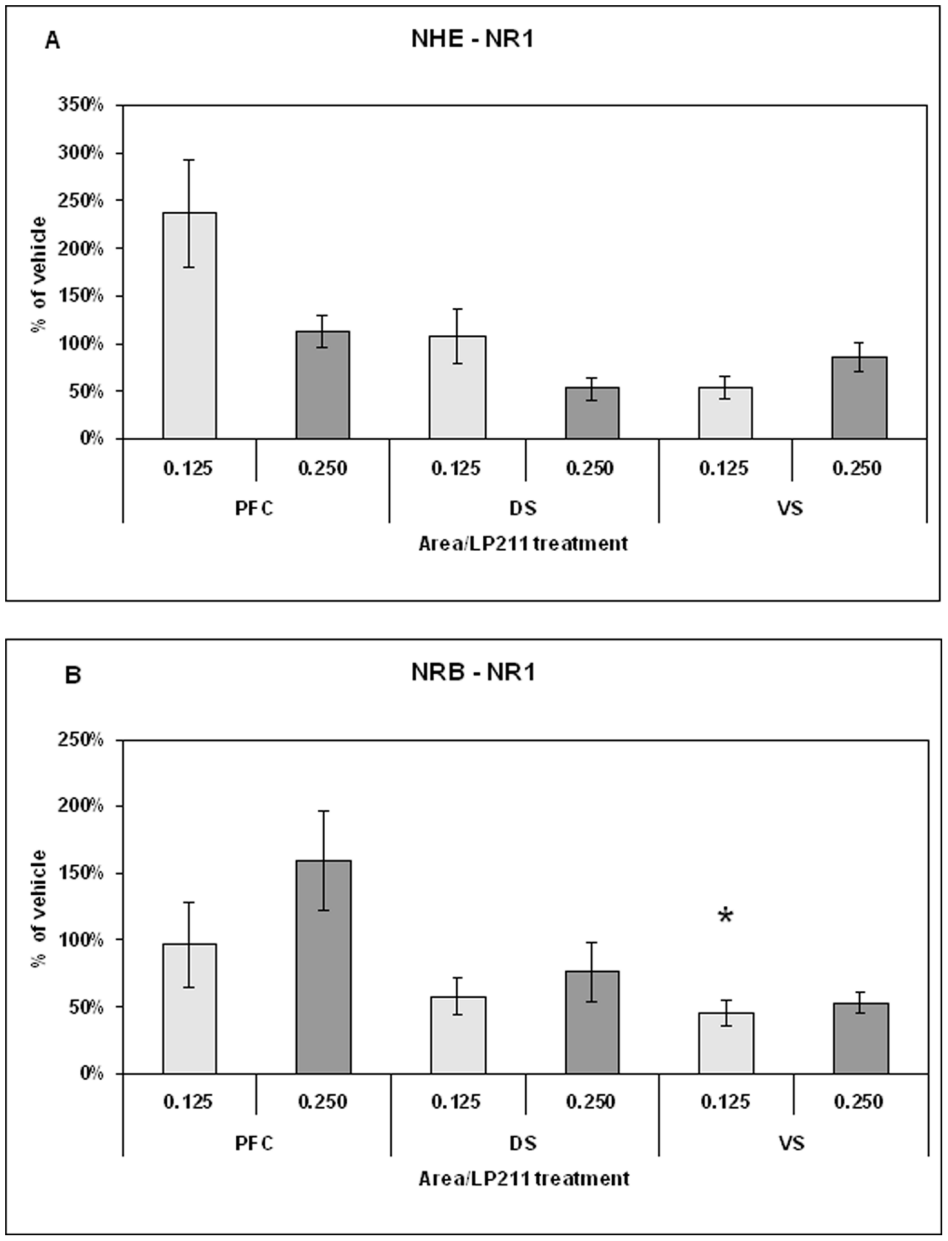
Level of NMDAR1 protein measured by western blot in the forebrain sites of NHE (panel A) and NRB (panel B) rats after adolescent treatment with 0.125 or 0.250 mg/kg LP-211. Data are presented as % of vehicle response ± standard error. For statistical analysis see the Results section (* p<0.05).

**Figure 8:**
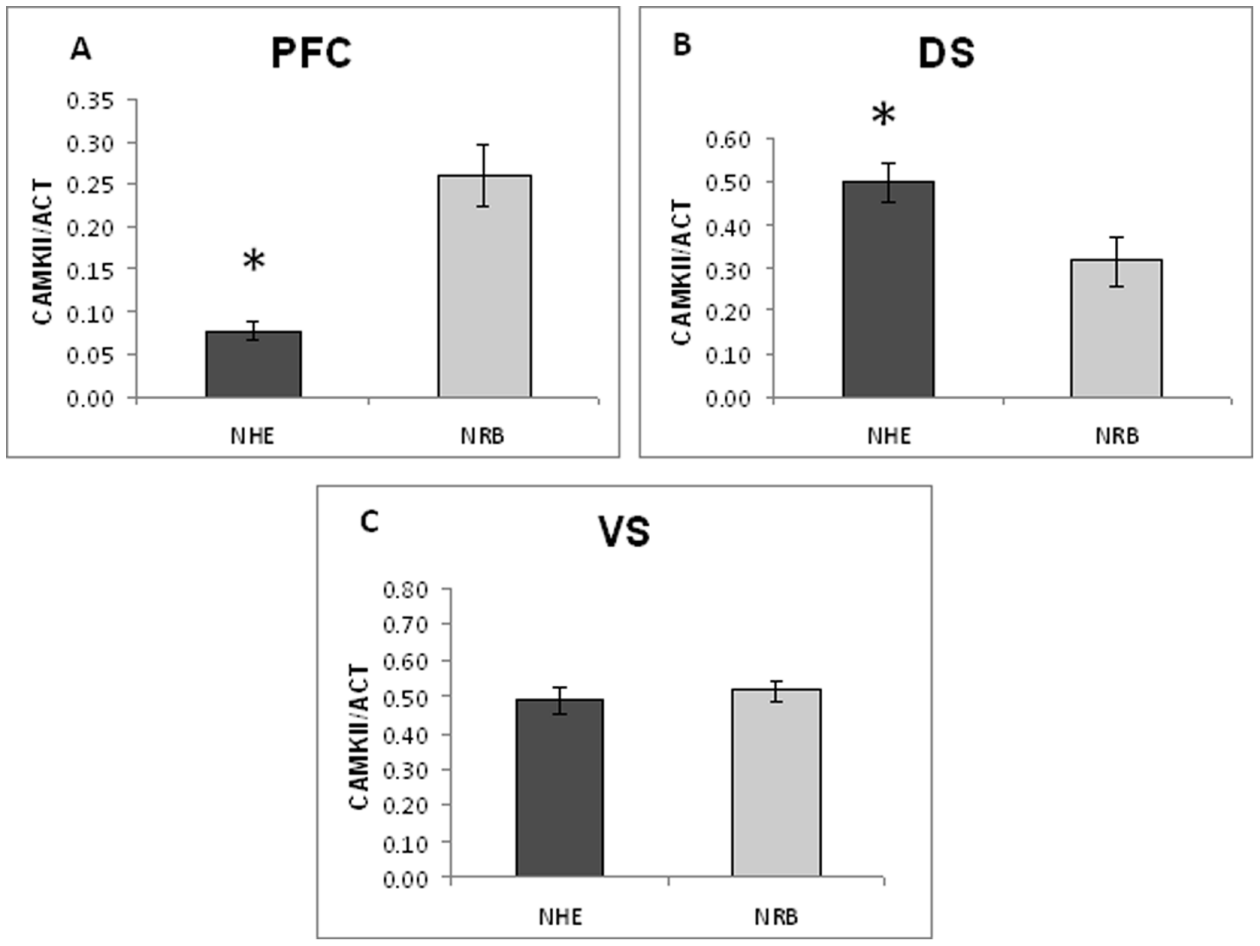
Level of CaMKIIα protein measured in the PFC (panel A), DS (panel B) and VS (panel C) of NHE and NRB rats under basal conditions. Data are presented as mean ± standard error. For statistical analysis see the Results section (* p<0.05).

**Figure 9:**
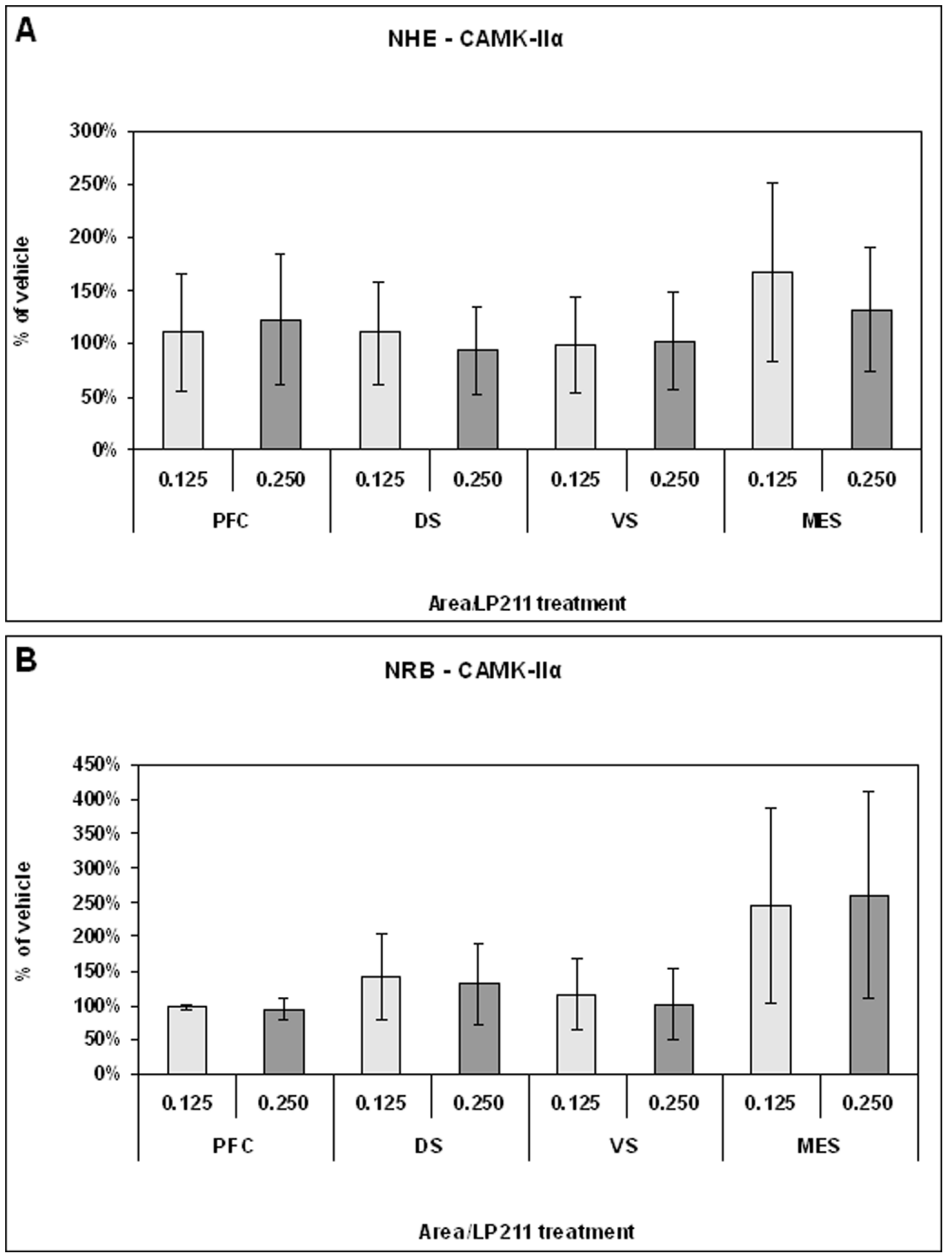
Level of CaMKIIα protein measured by western blot in NHE (panel A) and NRB (panel B) rats after adolescent treatment with 0.125 or 0.250 mg/kg LP-211. Data are presented as % of vehicle response ± standard error.

Long term changes in neural substrates were detected using, as molecular probes, the synaptic transmission markers dopamine transporter (DAT), NMDAR1 subunit and calcium-calmodulin-dependent kinase type II alfa (CaMKIIα). DAT has been chosen as presynaptic marker [25], [26], NMDAR1 as postsynaptic marker [27], whereas CaMKIIα served as an intracellular coincidence detector device [28]. Data pertaining to specific brain areas are shown in Figures 5, 6, 7, 8, and 9.

#### Ex-vivo levels of DAT protein

In the PFC, the one-way ANOVA on the DAT level of NHE rats, treated with vehicle, 0.125 mg/kg or 0.250 mg/kg LP-211, showed a significant reduction at 0.125 mg/kg dose (F = 14.02; df = 1/9; p<0.006) (Figure 5A). This reduction was about 20% of the vehicle control. The dose of 0.250 mg/kg had no significant effect. Conversely, no significant difference was found in the PFC of NRB rats (Figure 5B). In the DS, separate one-way ANOVAs showed no significant effects due to treatment on DAT level of NHE and NRB rats (Figure 5A–B). In the VS, the one-way ANOVA on DAT level of NHE rats, treated with vehicle or LP-211, showed a significant treatment effect only at the 0.250 mg/kg dose (Figure 5A). In fact, there was an increase in DAT protein level (F = 14.39; df = 1/10; p<0.004) that accounted for 100%. In contrast, no significant differences were observed again in NRB rats (Figure 5B). Finally, within the Mesencephalon (ME), the separate one-way ANOVAs, on DAT level for NHE and NRB rats, showed no significant effect due to prepuberal treatment with vehicle or LP-211 (Figure 5A–B).

#### Ex vivo levels NMDAR1 protein

The goal of these experiments was to verify whether the higher density of the NMDAR1 subunit, found in the PFC of NHE rats, could be modified by stress-associated vehicle injections and/or LP-211 treatment at adolescence. NMDAR1 protein level in the PFC of NHE and NRB rats, under basal conditions, are shown in Figure 6A. Separate one-way ANOVAs showed a higher NMDAR1 level in NHE rats (F = 10.10; df = 1/7; p<0.019). This increase was about 75% compared to the NRB controls. A higher level of NMDAR1 was also found within the VS (Figure 6B) of NHE rats, but one-way ANOVA showed no significant difference versus controls.

NMDAR1 protein levels in the brain areas (PFC; DS; VS) of NHE rats, treated with vehicle, 0.125 mg/kg or 0.250 mg/kg LP-211, are shown in Figure 7A. The separate one-way ANOVAs revealed no significant differences for NMDAR1 levels in the PFC, the DS and the VS of NHE rats, and this for all LP-211 treatment dosages. No significant changes were identified from the analysis of the PFC and DS data from NRB rats (Figure 7B). In the VS of NRB rats, treated with LP-211 at 0.125 mg/kg dose, the protein level was about 45% of that found in the vehicle-treated controls (F = 18.65; df = 1/8; p<0.003). A very similar but not significant reduction was observed for the 0.250 mg/kg dose.

#### Ex-vivo levels of CaMKIIα protein

The spatio-temporal coincidence detector CaMKIIα [28] was chosen for its integrator function in the intracellular traffic signalling, in relation to complex behaviours. Therefore, LP-211 treatment could modify this molecular device. CaMKIIα protein levels were analysed by western blot in the forebrain of NHE and compared to NRB rats, to determine the long-term effect of the adolescent treatment with vehicle, 0.125 mg/kg or 0.250 mg/kg LP-211 (Figure 8). Separate one-way ANOVAs on NHE and NRB rats showed, under basal conditions, a lower level of CaMKIIα in the PFC (F = 6.19; df = 1/7; p<0.042) of NHE compared to NRB rats (Figure 8A). This reduction accounted for 29.6% of the NRB level assumed as 100%. By contrast, an increased protein level was observed in the DS (F = 6.13; df = 1/11; p<0.033; Figure 8B). No significant differences were observed in the VS (Figure 8C). Long lasting effects of LP-211 prepuberal treatment were not observed in NHE and NRB rats (Figure 9A and B, respectively).

## Discussion

The rationale for these studies was to investigate whether and to what extent a prepuberal subchronic stimulation of serotonin 7 subtype receptors (5-HT7-R), by the selective LP-211 agonist, would produce long term effect on adult behaviour, amino acid transmitters and synaptic markers. This was carried out using a validated animal model of hyperactivity and attention deficit, the NHE line, and compared to the NRB control rats.

A prepuberal subchronic design was chosen based on the assumption that i) the prepuberal period is “critical” for the organisation of neural networks in the developing forebrain, and that these sites are sensitive to genotype x environment interactions [29]; ii) that repeated administration of a 5-HT7-targeting agonist would trigger a cascade of events leading eventually to enhanced neural plasticity [30]–[32].

By using a multi-disciplinary approach, we show that adolescent exposure to LP-211 yields long lasting changes on adult behaviour, improving spatial attention in a dose dependent manner, associated with modified pre/post synaptic markers.

### Long term behavioural effects persisting into adulthood

Prepuberal subchronic treatment apparently modified neural circuits underlying locomotor activity, NSA and SSA, as monitored in adulthood by exposure to two spatial novelty paradigms (i.e. Làt and Olton mazes). Under basal conditions, the NHE rats showed a higher frequency of leanings than NRB rats [14]. In NHE rats, 0.125 mg/kg LP-211 during adolescence reduced adult horizontal activity. The sub-chronic prepuberal treatment with the intermediate 0.250 mg/kg LP-211 dose produced significantly improved SSA that persisted into adulthood, even though it had no long-term effect on the frequency of rearings and leanings. The performance of these animals in the Olton's maze was almost perfect, indicating enhanced attention to spatial information and suggesting improved hippocampal function. The LP-211 treatment of adolescent rats has been recently demonstrated [33] to rearrange forebrain networks, with a central role of hippocampal connections, a finding which in turn confirms the emerging importance of 5-HT7 receptors for learning and memory processes. Thus, results are consistent with previous reports in rats [13], [34].

In particular, the stimulation of 5-HT(7) receptors could alternatively have been induced by a mixed 5-HT(1A/7) agonist with the help of a 5-HT(1A) selective antagonist [35]–[37]. This classic approach has been presently overcome by the availability of a direct 5-HT(7) agonist. It has been suggested that 5-HT(7) stimulation impairs short-term memory while improving long-term memory, and that acquisition requires silencing of 5-HT(7) while consolidation requires its activation [13], [34], [38]. For instance, 5-HT(7) antagonists are able to reverse a working memory deficit [39]. No effect has been reported under our experimental conditions (of prepuberal subchronic LP-211 exposure) on anxiety level, as monitored by elevated Zero-maze in the NHE rat line [40]. Therefore, we hypothesize that the beneficial effect of LP-211 on SSA might be due to enhanced attentive processes/mechanisms rather than to reduced emotionality. The absence of an effect by LP-211 on behavioural variables in the Làt maze, and the reduced horizontal activity in the Olton's maze, might be tentatively explained by the differential effect of 5-HT(7) stimulation on egocentric versus allocentric spatial mapping [38]. Indeed, spatial mapping in these two paradigms reveals a different neural mechanism: egocentric in the Làt maze and allocentric in the Olton's maze [41]. Thus, we have identified a second significant effect on spatial orientation, in addition to that induced previously by intranasal dopamine (INDA) treatment with the 0.150 mg/kg dose [42].

### Long term effects on amino acids and synaptic markers into adulthood

#### Excitatory amino acids

The ratio of Glutamate and Aspartate over Leucine, an amino acid not involved in neuro-transmission, pertained only to the soluble forms and was comparable to that measured in micro-dialysis [24]. This was presently an attempt to study, within *ex vivo* tissues, the compartmentalisation of excitatory amino acids in the mammalian brain. The soluble form includes amino acids that can be washed out with cold saline together with part of the neuro-glial component. Amino acids are distributed in three main heterogeneous pools, for which a homeostasis has been proposed by Kalivas [43]. These include a very large metabolic pool (10 milliMoles) of neuro-glial origin, a smaller vesicular pool and an even smaller extra synaptic pool [44]. In addition, they are not independent as L-Glu, first stored in the glial pool, is transferred to the neuron as glutamine, then is deaminated back to L-Glu and finally packed into vescicles to be secreted into the synaptic cleft. Therefore, and even though the metabolic compartment overwhelms the others by its size (10 mM), the higher level of L-Glu should be proportionally distributed among the three pools. On the other hand, L-Asp was measured, as mentioned in the Introduction, because of its hypothesised role in the formation of NMDA molecules, which are agonists at the NMDA receptor sites. Therefore, L-Asp data should be considered in conjunction with NMDAR1 binding sites (see below). We predict that the beneficial effect of adolescent LP-211 is due to altered amino acid transmission. This, in turn, may prevent glutamate neurotoxicity - that imaging studies have shown to be the basis for right fronto-striatal atrophy in ADHD [45], [46]. Adolescent exposure to LP-211 yielded indeed two major consequences: i) it decreased L-Glu and L-Asp in the DS after treatment with the 0.125 mg/kg dose and ii) it increased L-Glu in PFC and L-Asp in the VS after treatment with the 0.250 mg/kg dose, both in the NHE rats. L-Glu also decreased, after prepuberal subchronic treatment with 0.125 mg/kg LP-211, only in the VS of control NRB rats. Thus, prepuberal exposure to serotonergic stimulation permanently modifies neural areas and forebrain circuits underlying activity, motivation and attention, but not emotionality. By focussing on the prefronto-striatal interface, the most relevant change was produced by adolescent exposure to 0.250 mg/kg LP-211, with persistent amino acid changes found in both PFC and VS of an animal model for hyperactivity and attention deficit.

#### Synaptic markers

The presynaptic DAT, the post-synaptic NMDAR1 and CaMKIIα markers, studied under basal conditions, showed that NMDAR1 levels within the PFC of NHE rats were higher, in comparison with NRB rats; by contrast, no differences were found within the DS. Moreover, NHE rats showed a significant reduction in the amount of CaMKIIα within the PFC, while previous findings [47], [14] have shown that DAT level within the PFC is higher for NHE. Conversely, an increased level of CaMKIIα was detected in the DS while the VS did not show any difference between NHE and NRB rats. Finally, the level of CaMKIIα in the mesencephalon - though very low [48] - was found to be higher in NHE rats.

Treatment with LP-211 during adolescence gave the following findings that persisted into adulthood: 1) DAT level in the PFC was decreased at the 0.125 mg/kg dose and conversely increased in the VS at the 0.250 mg/kg, but only in NHE rats. The adolescent prepuberal treatment produced no differences in all areas of NRB control subjects; 2) NMDAR1 was decreased in the VS of NRB rats when previously faced with the prepuberal subchronic exposure to a 0.125 mg/kg dose; 3) prepuberal subchronic LP-211 exposure exerted no long-term effect on CaMKIIα levels in either line and in all areas. It is noteworthy that CaMKIIα level has been reported to be lower in the forebrain of animal models for ADHD, such as SHR and NHE rats [49]–[51].

Altogether, the most relevant change was produced by the 0.250 mg/kg dose of LP-211: adolescent exposure to this dose generated, as a sequel into adulthood, an improved spatial orientation as well as persistently elevated L-Asp and DAT levels in the VS and L-Glu levels in the PFC. The simplest interpretation of these results is that the cross-talk between the PFC and VS might have been improved.

### Prefronto-striatal interface in model systems

The “dialogue” between the PFC and both the DS and VS, occurring through glutamate and aspartate, is modulated mainly by DA, NA, 5-HT and histamine. Our experimental evidence suggests that only two signaling modulators (DA and 5-HT) are effective in inducing long-term changes within neural networks, which are known to underlie attentive and motivational processes. In fact, prepuberal subchronic treatments with MPH and ATX, which block the reuptake of DA/NE and NE, respectively, did not similarly alter the SSA in adult rats, in spite of significant effects on DA, NA, 5-HT and metabolites at the prefronto-striatal interface in NHE rats [52]. By contrast, we found previously that an INDA-based prepuberal subchronic treatment is effective at a low dose (0.075 mg/kg) [53].

This profile of effects is likely to be explained because DA has the following functions: i) at mesencephalic level, DA can interact with DA-D2 autoreceptors, associated with hyper-polarising potassium conductances, thereby reducing the firing rate of DA neurons [54] and ii) at 0.150 mg/kg dose, DA can induce the synthesis of neurotrophic factors of the BDNF type [55]. On the other hand, the MPH-induced DA increase, caused by MPH itself and ATX, clearly suggests that the DA signal is much more effective in the absence of other modulator signals.

The fact that the developmental stimulation of 5-HT7 receptors (by the selective agonist LP-211) is just as effective as DA-related drugs in modifying the long-term SSA in NHE rats requires further experimental investigations. One clue is given by the fact that 5-HT7 is found to be functionally linked to forebrain limbic networks, which comprise the amygdala, VS, orbital PFC, and thalami [33]. Moreover, a recent report [56] demonstrated that serotonin acts on inhibitory interneurons in the PFC through Gs proteins. The PFC-mediated inhibitory control over subcortical structures might well be mediated by 5-HT7 receptor via its basal level of activation and possibly through neuroplastic processes [57].

Therefore, we can assume that the positive effect of adolescent exposure to 0.250 mg/kg LP-211 on SSA, in association with enhanced levels of amino acids and DAT in the PFC and VS, is possibly due to the stimulation of serotonergic more effective transducers independent of CaMKIIα. Alternatively, this positive effect might be explained by more complex circuits, leading - eventually - to the re-modeling of cognitive maps: in this context, recent findings [33] point to enhanced transmission between limbic networks including the hippocampus, that might explain the improved SSA (essential to build spatial maps). Furthermore, the same approach has confirmed the hypothesis that the mesocortical DA branch is, in the NHE rat model, at a higher functional state [14], [16], [17]. In addition, the subchronic treatments using other selective agonists of the 5-HT7 receptors, during the prepuberal period, may induce medium/long term effects on selective spatial attention, underpinned by synaptic markers such as DAT, NMDAR1 but not CaMKIIα.

Finally, the concentration of CaMKIIα [28], which is a coincidence spatio-temporal detector device, was reduced in the PFC of NHE rats under basal conditions and increased in the DS as compared to NRB. Interestingly, the reduced CaMKIIα levels in the PFC were associated with a reduced spatial orientation of the NHE rats in the Olton's maze. On the other hand, adolescent exposure to LP-211 did not change the level of CaMKIIα in the forebrain, but significantly improved the SSA. In fact, as mentioned in the Introduction, CaMKIIα is a molecular device that integrates neural signals at higher complexity levels, as demonstrated by Yovell and Abrams [58]. They studied the phosphorylation of the Aplysia membrane proteins by this kinase in the presence of serotonin. The CaMKIIα protein is localised mainly at post-synaptic level, but it is also present at pre-synaptic levels. The addition of calcium and serotonin within 8 sec significantly increased the phosphorylation of the Aplysia membrane proteins by the kinase, thereby demonstrating a classical Pavlovian conditioning in a test tube [58], [59].

Interestingly, recent findings from our laboratory do point to a disintegrated interface in the PFC of NHE rats, as monitored by immuno-chemical markers [60]. This, in turn, might explain a functional “vulnus” in the default mode network that includes the medial PFC, anterior cingolate, as well as lateral-parietal and temporal cortices, as proposed in [61]–[65].

## Materials and Methods

### Animals

Prepuberal male rats of the Sprague–Dawley-derived NHE line (n = 24) and of the NRB control line (n = 24), from our animal colony, were used throughout the experiments. Rats were housed in groups of two inside Makrolon cages (425 mm×266 mm×150 mm, floor area 820 cm^2^; Techniplast, Italy) under standard conditions. An artificial 12∶12 reverse light–dark (LD) cycle was obtained by turning on the lights from 7 pm to 7 am. All other parameters fulfilled the requirements of the Guide for the Care and Use of Laboratory Animals, implemented by the Italian Legislative Decree 116/1992 that covers all scientific procedures involving the use of live animals. The European Directive 2010/63 on the protection of animals used in scientific experiments, revisioning the Directive 86/609, is currently being enacted into Italian legislation, and will be applicable to future research protocols. All present experiments were covered by an authorisation from Ministero della Salute, for which ethical advice was issued from Istituto Superiore di Sanità (authorization to AGS, n. prot. 422/2009 dated 08-04-09, duration 36 months). The 3Rs principles were taken into account in order to seek for alternatives to animal use, to reduce the number of animals used and to refine the protocols for limitation of animal suffering.

### Prepuberal treatment

At weaning on postnatal day (pnd) 28, rats were randomly assigned to treatment and control groups (n = 6/group), which received i.p. vehicle or LP-211 (0.0, 0.125, 0.250 or 0.500 mg/kg). LP-211 was dissolved in phosphate buffer (vehicle) with 1% and 2% DMSO for the lower doses and the highest one, respectively. Treatments were given daily during the dark phase between 10:00 am and 11:00 am, for 14 days from pnd 30–31 to 43–44, as already published [40]. Thirty days after last drug or vehicle injection, all the rats were tested in the Làt maze and, 24 hrs later, in the eight-arm radial maze (Olton's maze).

### Drugs

A new selective 5-HT7 receptor agonist, namely N-(4-cyanophenylmethyl)-4-(2-diphenyl)-1-piperazinehexanamide (LP-211), was synthesized and provided by EL and ML [12]; drug dosage was chosen according to previous experience [40] by GL and WA. DMSO was obtained from Sigma-Aldrich (Milano, Italy).

### Làt-maze

#### Apparatus

The Làt-maze consisted of a 60 cm×60 cm×40 cm box made of special poly-vinyl-chloride (PVC) material (KÖMACEL^R^), closed by a cover of the same material. A smaller transparent-plastic 30 cm×30 cm×40 cm box was inserted in the middle, thus providing a 60 cm long, 15 cm wide and 40 cm high square-shaped exploratory corridor. See [66] for details. The box was illuminated by a white, cold 4 W lamp placed 60 cm above the floor in the centre of the cover, providing 0.1–0.2 µW/cm^2^. A set of two such boxes was located in a sound attenuated experimental room.

#### Procedure

At the beginning of the dark phase of an inverted light/dark L/D cycle (between 9 am and 4 pm), the rats were individually exposed to the Làt-maze and allowed to explore the corridor for 10 min. In order to minimise the interference with the arousal state, pairs of rats from the same home cage were tested at the same time. The animals' behaviours were monitored by a high resolution CCD camera and stored on a DVD recorder to be analyzed off-line. The number of corner crossings (HA), frequency (VA) and duration (RD) of rearings on hind-limbs, or leanings against the walls with one or both forepaws, were measured in 1-min blocks. The reliability index was quite high (r = 0.914; df = 198; p<0.001). At the end of the test, the fecal boluses were counted and the floor was wiped with a wet sponge.

### Eight-arm radial maze (Olton's maze)

#### Apparatus

The apparatus consisted of eight arms (8 cm×60 cm) extending from an octagonal centre platform (18.5 cm diameter). The distance from the platform centre to the end of each arm was 69.25 cm. The apparatus was constructed of grey PVC with a smooth surface, and side walls (14 cm high, transparent Plexiglas). The maze was placed on the floor of a dimly lit room, surrounded by a circular higher wall without visual cues. Rat behaviour was monitored by a high resolution CCD camera and stored on DVD to be analysed off-line.

#### Procedure

In the Olton's maze, each rat was placed on the centre platform into a cardboard cylinder to avoid immediate escape into an arm. The trial began with the removal of the cylinder and the rat was allowed to explore. The main parameters considered in the Olton's maze were horizontal activity (HA: number of alley visits); frequency (VA) and duration (RD) of rearings on the hind-limbs. The number of arms visited before the first repetition occurred (first entry, FE), and number of arms visited before completion of visits to all eight arms (number of visits to criterion, NVTC), were also recorded. The latter two indices are markers of non-reinforced SSA.

### Dissection of brain areas

The animals were sacrificed by a guillotine at pnd 77. The brain was removed and put on a surface of ice-cold saline. After removal of the olfactory tubercles, the first coronal cut was made at 4.20 AP from Bregma, using the stereotaxic coordinates of *The Rat Brain Atlas* as reference [67]. By this method, a tiny tissue included both PFC and cingulate cortex area 1. The entire upper portion of the striatum (i.e. DS), which included the caudate-putamen (CPu) and globus pallidus (CG), was removed by a sagittal pinch extending between 2.20 and −3.8 AP, up to 7 mm DV in depth. Then, ventral striatum (i.e. VS) was removed by sagittal pinch between 2.70 to 0.48 AP, 1 mm from the midline and about 1 mm in depth, thus including the nucleus accumbens.

### Amino acids

#### Extraction procedure

Vehicle, LP-211 0.125 and 0.250 mg/kg groups were analysed. The 0.500 mg/kg group was excluded because it did not show any significant effect in the behavioural analysis. Brain samples were homogenised in 1 ml ice-cold saline in an Eppendorf and centrifuged at 7,500 g at 4°C for 20 min. The supernatant was filtered and used for LC/MS/MS analysis of the free amino acids L-Glu, L-Asp and L-Leu.

#### Liquid chromatography/tandem mass spectrometry

The detection was performed using a Varian 310-MS triple quadrupole mass spectrometer (Varian, Palo Alto, CA, USA). All the analytes were detected in the positive ionisation mode and with selected reaction monitoring (SRM) mode (Table 1). The settings of the ESI source were as follows: spray voltage, 5000 V; capillary temperature 300°C; sheath gas pressure (spraying), 20 arbitrary units; auxiliary gas pressure (desolvating), 10 arbitrary units; and ion sweep gas pressure (curtain), 5 arbitrary units. The collision cell (Q2) pressure was 2.2 mTorr of argon. The collision energies were optimised for maximum detection of each product ion (Table 1). Chromatographic separation was achieved with a ProStar™ 300 HPLC system (Varian, Palo Alto, CA, USA) on a Varian Polaris® C_18_column (5 µm, 2.1 mm×100 mm) at a flow rate of 0.3 mL/min. The mobile phase consisted of aqueous 0.1% formic acid (A) and acetonitrile (B). Samples were eluted with a linear gradient from 10 to 90% B in 5 min. At 5:01 min, solvent B was decreased from 90% to 10% and remained constant for 5 min. The total run time was 10 min.

**Table 1:** LC/MS/MS parameters.

Analyte	RT (min)	SRM transitions *(m/z)*	CE (eV)
Aspartate	1.36	134.0→134.0	4.0
		134.0→116.0	4.0
Glutamate	1.38	148.0→148.0	4.0
		148.0→130.0	7.5
Leucine	1.42	132.2→132.2	4.0
		132.2→86.0	10.0

### CaMKIIα, DAT, and NMDAR1

#### Protein extracts

Membrane and cytosolic protein fractions were prepared as described in [68] with minor modification. Tissues were homogenized in a glass/Teflon potter in cold 0.32 M sucrose buffer pH 7.4 containing 1 mM HEPES, 0.1 mM EGTA and 0.1 mM phenylmethylsulfonyl fluoride, in the presence of commercial cocktails of protease (Roche, Monza, Italy) and phosphatase (Sigma-Aldrich) inhibitors. The homogenate was centrifuged at 1,000 g for 10 min resulting in a pellet (P1) that corresponded to the nuclear fraction. The supernatant (S1) was then centrifuged at 13,000 g for 15 min to obtain a clarified fraction of cytosolic proteins (S2) and a pellet (P2) corresponding to the crude membrane fraction. The latter was homogenised in a glass/Teflon potter in 1% Triton X-100 buffer containing 50 mM Tris-HCl pH 7,4–300 mM NaCl, 5 mM EDTA and 0,02% Sodium Azide. The total protein content was measured according to the Bradford Protein Assay procedure (Bio-Rad, Milan, Italy), using bovine serum albumin as the calibration standard.

#### Gel electrophoresis and immunoblotting

Sodium dodecyl sulfate-polyacrylamide gel electro-phoresis (SDS-PAGE) and transfer of proteins to nitrocellulose membranes were performed according to conventional methods with minor modifications. The proteins were solubilised in loading buffer at 54°C for 45 min, and then separated on 10% polyacrylamide gels and transferred onto PVDF membranes (GE Healthcare) in transfer buffer (25 mM Tris, 192 mM glycine, pH 8.3, 20% methanol). The membranes were incubated with blocking buffer, 5%ECL blocking agent (GE Healthcare) in PBST (0.1 M phosphate buffered saline, pH 7.4, 0.1% Tween-20), for 1 h at room temperature. The blots were incubated overnight at 4°C with one of the following primary antibodies: NMDAR1 (1∶300 anti-NMDAR1; Chemicon-Millipore AB9864); CaMKIIα (1∶2000 Anti-CaM Kinase IIα, Clone 6G9; Upstate-Millipore #05-532); DAT (1∶500 anti-Dopamine Transporter; Millipore AB2231); internal standard: Actin (1∶500 anti-Actin; Sigma A2066); or Actin (1∶3000 anti–Actin, Clone C4; Chemicon-Millipore MAB1501).

After three 10 min washes in PBST, the blots were incubated for 1 h at room temperature with horseradish peroxidase-conjugated secondary antibody (anti-rabbit or anti-mouse ECL anti-rabbit IgG, HRP-linked Whole Ab; GE Healthcare NA934, NA931). After washing, immuno-complexes were visualised by chemiluminescence using the ECL western blotting kit (GE Healthcare) according to the manufacturer's instructions. All protein bands were within linear range of the standard curves, and were normalised to actin levels on the same membrane. Quantity One software (BioRad Laboratories, Hercules, CA, USA) was used for standardisation and quantisation of protein bands obtained by western blot analysis.

### Statistics

The fulfilment of the requirements for parametric analysis (normality, variance, etc.) was assessed by specific tests (Kolmogorov–Smirnov and Levene, Box test of equality of covariance matrices) and appropriate transformations were used when necessary. Behavioural variables in the Làt and Olton's radial maze were submitted to a one-way factorial analysis of variance (ANOVA) for treatment followed by post-hoc test LSD for single comparison.

Defecation scores were submitted to a non-parametric test by Kruskal–Wallis. The rejection level was set at *p*>0.05 after correction of the alpha-level by the Hölm's procedure [69]. All statistical analyses on behavioural and neurochemical data were performed by SPSS software. All data are stored on a hard disk of a PC in our laboratory and a back-up copy in also saved in a mobile HD unit. They will be kept at least for the next ten years and can be made available on request.
